# Prostate Cancer Risk Is not Altered by *TP53AIP1* Germline Mutations in a German Case-Control Series

**DOI:** 10.1371/journal.pone.0034128

**Published:** 2012-03-23

**Authors:** Manuel Luedeke, Irina Coinac, Carmen M. Linnert, Natalia Bogdanova, Antje E. Rinckleb, Mark Schrader, Walther Vogel, Josef Hoegel, Andreas Meyer, Thilo Dörk, Christiane Maier

**Affiliations:** 1 Department of Urology, University Hospital Ulm, Ulm, Germany; 2 Institute of Human Genetics, University Hospital Ulm, Ulm, Germany; 3 Radiation Oncology and Gynaecology Research Unit, Hannover Medical School, Hannover, Germany; IFOM, Fondazione Istituto FIRC di Oncologia Molecolare, Italy

## Abstract

Prostate cancer susceptibility has previously been associated with truncating germline variants in the gene *TP53AIP1* (tumor protein p53 regulated apoptosis inducing protein 1). For two apparently recurrent mutations (p.Q22fs and p.S32X) a remarkable OR of 5.1 was reported for prostate cancer risk. Since these findings have not been validated so far, we genotyped p.Q22fs and p.S32X in two German series with a total of 1,207 prostate cancer cases and 1,495 controls. The truncating variants were not significantly associated with prostate cancer in none of the two cohorts, nor in the combined analysis [odds ratio (OR) = 1.16; 95% confidence interval (CI 95%) = 0.62–2.15; p = 0.66]. Carriers showed no significant differences in family history of prostate cancer, age at diagnosis, Gleason score or PSA at diagnosis when compared to non-carrier prostate cancer cases. The large sample size of the combined cohort rejects a high-risk effect greater than 2.2 and indicates a limited role of *TP53AIP1* in prostate cancer predisposition.

## Introduction

Prostate cancer (PrCa) is the most frequent tumor and the third leading cause of cancer related death in men in the western industrial world [1]. In the etiology of PrCa a considerable degree of heritability is assumed to be involved [2]. To date, more than 30 moderately risk-associated single nucleotide polymorphisms have been identified in genome wide association studies, which explain a minor fraction of heritability [3]. In contrast, high risk genes which could be responsible for the majority of the observed familial clustering are still unknown.

There is growing evidence that genes involved in DNA damage response play a predisposing role for PrCa [4]. This includes DNA repair processes as well related mechanisms like apoptotic pathways. TP53AIP1 (tumor protein p53 regulated apoptosis inducing protein 1) plays a key role in the tumor suppressor p53 dependent apoptotic signaling. TP53AIP1 is localized in the mitochondrial membrane and mediates apoptosis through cytochrome *c* release [5]. The expression of *TP53AIP1* is triggered upon severe damage by phosphorylation of p53 at Ser46 [6]. *TP53AIP1* was found to be mutated in PrCa tissue in a recent study [7]. The two identified truncating mutations p.Q22fs and p.S32X turned out to be recurrent germ-line variants and were strongly associated with PrCa risk (OR of 5.1), thus suggesting *TP53AIP1* as a promising susceptibility gene. In the present study we have evaluated the risk effect of these two *TP53AIP1* mutations in two independent German PrCa cohorts.

## Results

Both truncation variants were observed in our cohorts. While the frameshift allele p.Q22fs was present in 1.8% cases and 1.6% controls, the nonsense mutation p.S32X appeared to be comparably rare (0.1% carriers in both cases and controls). Overall, p.Q22fs and p.S32X were not associated with prostate cancer risk, neither in the combined series of Ulm and Hannover, nor in each individual sample (Table 1). No evidence against homogeneity was observed among the Ulm and Hannover series (p = 0.39). Accumulation in familial prostate cancer cases could indicate a high risk effect mediated by the variants. This hypothesis was tested in the Ulm series, which contained a collection of PrCa cases with positive family history. However no enrichment of the mutations was observed in familial cases (6 out of 377 (1.6%)) when compared to sporadic cases (7 out of 325 (2.1%)). Finally, clinical subgroups of cases were defined in order to elucidate trends towards aggressive tumor forms. The mutation carriers showed no significant difference from non-carriers in severity of prostate cancer, as determined by age of onset, Gleason score, or PSA level prior treatment (Table 2).

**Table 1:** Genotype specific odds ratio (OR), exact 95% confidence interval (CI 95%) and p-values of truncating *TP53AIP1* variants in case control comparisons.

		genotype, *n* (%)					
	*n*	p.Q22fs het^a^	p.S32X het^a^	p.Q22fs or p.S32X het^a^	normal(wild-type)	OR (CI 95%)	p-value
all controls	1495	24 (1.6%)	1 (0.1%)	25 (1.7%)	1470 (98.3%)	ref.	
all cases	1207	22 (1.8%)	1 (0.1%)	23 (1.9%)	1184 (98.1%)	1.16 (0.62–2.15)	0.66
controls Ulm	995	18 (1.8%)	1 (0.1%)	19 (1.9%)	976 (98.1%)	ref.	
cases Ulm^b^	702	13 (1.9%)	0 (0%)	13 (1.9%)	689 (98.1%)	0.97 (0.44–2.08)	1.00
controls Hannover	500	6 (1.2%)	0 (0%)	6 (1.2%)	494 (98.8%)	ref.	
cases Hannover	505	9 (1.8%)	1 (0.2%)	10 (2.0%)	495 (98.0%)	1.66 (0.54–5.61)	0.45

a : heterozygous.

b : including 377 familial and 325 sporadic prostate cancer patients.

**Table 2:** Comparison of age at diagnosis, Gleason Score and PSA at diagnosis between carriers of the truncating *TP53AIP1* variants and non-carriers.

	p.Q22fs or p.S32X het^a^		normal (wild-type)		
	*n* b	mean (SD)	*n* b	mean (SD)	p-value
age at diagnosis	23	63.5 (±6.7)	1174	64.4 (±6.7)	0.53
Gleason Score	20	5.9 (±1.1)	1009	6.1 (±1.1)	0.42
PSA at diagnosis [ng/mL]	21	12.2 (±10.3)	1087	13.6 (±53.0)	0.91

a : heterozygous.

b : probands with available information for specified parameter.

## Discussion

*TP53AIP1* has been suggested as a PrCa susceptibility gene, since two recurrent germline mutations (p.Q22fs and p.S32X) were found considerably overrepresented among PrCa patients within a previous case-control study [7]. For the purpose of confirmation, we have genotyped p.Q22fs and p.S32X in approximately 1,200 prostate cancer cases and 1,500 controls, but we failed to replicate an association of these variants with PrCa risk.

The discrepancies between the present and the initial study could be explained by several factors, including variable ethnic backgrounds, different sample sizes and diverging inclusion criteria. Population-specific differences have become evident in the case of p.S32X, as this variant has been found rarely in German probands, and thus could not be evaluated for disease association. The most obvious difference between the study samples is the frequency of variant p.Q22fs, which appeared much lower in the control group of Wang and colleagues as compared to our controls (0.3% versus 1.6%, respectively). Noteworthy, Wang et al. have recruited healthy, age matched controls, which could be more powerful to detect associations with prostate cancer risk, despite of their 4.5-fold smaller sample size. In contrast, the unselected controls for our comparisons may contain misclassified prostate cancer cases at population prevalence levels, so that true disease effects would appear slightly understated. However, power estimations suggest that the loss of power by choosing population controls instead of “super controls” would be small for a disease with an about 10% incidence rate, and that this loss can be efficiently compensated for by modest increases in sample size [8]. The fact that we observed virtually equal carrier frequencies in both cases and controls strongly argues against an association of p.Q22fs with PrCa. Based on the sample sizes enrolled here, a resulting interval of confidence rules out any risk effect greater than 2.2 for p.Q22fs and p.S32X.

In subgroup analyses we have considered predisposing roles of *TP53AIP1* mutations particularly for familial and for aggressive PrCa. No accumulation of p.Q22fs and p.S32X was observed in familial cases, rejecting the hypothesis of a high penetrance of these mutations. Finally, the lack of genotype/phenotype correlation with respect to clinical parameters argues against susceptibility to more severe forms of PrCa for the mutation carriers.

Aside from truncating mutations examined in the present study, a missense substitution (p.A7V) in *TP53AIP1* has previously been studied in the Hannover series. Similarly, the p.A7V variant also had not shown an increased frequency in prostate cancer cases [9]. We conclude that there is little support at present to regard *TP53AIP1* as a prostate cancer susceptibility gene.

## Materials and Methods

### Probands

Two series of cases and controls were pooled for this study, recruited at the Universities of Ulm and Hannover, Germany. All probands were of Caucasian origin. The study was approved by the Institution Review Board of Ulm (Ethikkommission der Universität Ulm - vote number 87/97) and the Institution Review Board of Hannover (Ethikkommission der Medizinischen Hochschule Hannover - vote number 3894/05). Written informed consent, according to the Institution Review Boards, was obtained.

From the Prostate Cancer Genetics Project of Ulm a total number of 377 familial and 325 sporadic cases were included. The recruitment scheme of these patients is described elsewhere [10]. The median age of diagnosis was 63 years (range 40–84 years) and the majority was treated by radical prostatectomy. For association tests 995 population controls from Ulm were used.

The Hannover prostate cancer study (HaPCS) consists of a hospital-based series of 505 unselected patients with PrCa who were treated with brachytherapy between October 2000 and September 2007 at Hannover Medical School [9]. All patients had biopsy-proven adenocarcinoma of the prostate. Indication for permanent brachytherapy was clinically localized low risk early PrCa (cT2a or less with a PSA serum level <10 ng/ml and a Gleason score <7). The median age at diagnosis was 67 years (range 42–82 years). For comparison, 504 genomic DNA samples were collected from adult male blood donors at Hannover Medical School between 2006 and 2007.

### Genotyping

Genomic DNA, extracted from peripheral blood lymphocytes, served as template for genotyping. The Ulm cohort was typed for p.Q22fs with the high resolution melting (HRM) method. In brief: PCR was performed with AmpliTaq Gold Mastermix (Applied Biosystems, Foster City, USA). After PCR amplification 1.0 µL EVA-Green (Biotium, Hayward, USA) was added to each sample and the dissociation curve was measured on a 96-well 7900HT Fast Real-Time PCR System (Applied Biosystems, Foster City, USA). HRM data was analysed with the High Resolution Melt software v1.1 (Applied Biosystems, Foster City, USA). The S32X variant was typed using a Custom SNP Genotyping Assay (Applied Biosystems, Foster City, USA) along with TaqMan Genotyping Mastermix (Applied Biosystems, Foster City, USA) in a total volume of 5 µL on a 384-well 7900HT Fast Real-Time PCR System (Applied Biosystems, Foster City, USA). For positive controls we used plasmids corresponding to the truncating allele p.Q22fs or p.S32X respectively and the normal allele. Normal allele and mutant plasmids were mixed 1∶1 in order to mimic heterozygous genotypes. Genotyping of the Hannover series was performed with restriction fragment length polymorphism analyses using *Bgl*I for p.Q22fs and *Bfa*I for p.S32X, after PCR amplification. Primer and probe sequences, as well PCR conditions will be given on request. Samples that were identified to carry the p.Q22fs or p.S32X variant by any of the utilized screening methods were verified by Sanger sequencing.

### Statistical analyses

Associations between genotypes and disease status were assessed with SAS 9.2 (SAS, Cary, USA). Odds ratios and exact 95% confidence intervals are given, along with p-values by Fisher's exact test. For the combined analyses homogeneity of the odds ratios where checked by Breslow-Day test and the combined odds ratio was calculated with Mantel-Haenszel test. The unpaired T-test was used for comparison of clinical parameters in the mutation carriers and non-carriers and was calculated with StatView 5.01 (SAS, Cary, USA).
